# Submicroscopic malaria cases play role in local transmission in Trenggalek district, East Java Province, Indonesia

**DOI:** 10.1186/s12936-017-2147-7

**Published:** 2018-01-05

**Authors:** Heny Arwati, Subagyo Yotopranoto, Etik Ainun Rohmah, Din Syafruddin

**Affiliations:** 1 Department of Parasitology, Faculty of Medicine, Universitas Airlangga, Campus A, Jalan Prof. Moestopo No. 47, Surabaya, 60131 Indonesia; 2 Entomology Study Group, Institute of Tropical Diseases, Universitas Airlangga, Campus C, Jalan Ir. Soekarno, Surabaya, 60115 Indonesia; 3 Eijkman Institute for Molecular Biology, JalanDiponegoro 69, Jakarta, 10430 Indonesia; 4 Department of Parasitology, Faculty of Medicine, Hasanuddin University, Makassar, JalanPerintisKemerdekaan Km 10, Makassar, 90245 Indonesia

**Keywords:** Migrant workers, PCR positive malaria, Local transmission

## Abstract

**Background:**

Trenggalek district is a hypoendemic malaria area with mainly imported cases brought by migrant workers from islands outside Java. During malaria surveillance in 2015, no malaria cases were found microscopically, but some cases were positive by PCR. Therefore, a study was conducted to prove that local malaria transmission still occur.

**Methods:**

The adult villagers were invited to the house of the head of this village to be screened for malaria using aseptic venipuncture of 1 mL blood upon informed consent. Thin and thick blood films as well as blood spots on filter paper were made for each subject. The blood films were stained with Giemsa and the blood spots were used to extract DNA for polymerase chain reaction (PCR) amplification to determine the malaria infection. In addition, the history of malaria infection and travel to malaria endemic areas were recorded. Entomologic survey to detect the existence of anopheline vector was also conducted.

**Results:**

Of the total 64 subjects that participated in the survey, no malaria parasites were found through microscopic examination of the blood films. The PCR analysis found six positive cases (two *Plasmodium falciparum*, one *Plasmodium vivax* and two mixed infection of both species), and two of them had no history of malaria and have never travelled to malaria endemic area. Entomologic survey using human bait trap detected the existence of *Anopheles indefinitus* that was found to be positive for *P. vivax* by PCR.

**Conclusions:**

The results indicated that although we did not find any microscopically slide positive cases, six PCR positive subjects were found. The fact that 2 of the 6 malaria positive subjects have never travelled to malaria endemic area together with the existence of the vector confirm the occurence of local transmission of malaria in the area.

## Background

Malaria is caused by the protozoan genus *Plasmodium* and transmitted by female *Anopheles* mosquito. Malaria in Indonesia remains an important health burden. Indonesia has commited to eliminate malaria in the whole country by 2030, with phased target by region depending upon malaria endemicity [1]. East Java Province, a hypoendemic area in Java Island recorded 334 cases of imported malaria and three death of malaria during 2016, but no indigenous cases. The Trenggalek district has targetted to achieve malaria elimination in 2019 [2]. Until 2017, all districts except Trenggalek have been certified to be free from malaria. The inhabitants of Trenggalek frequently travel outside Java Island for temporary working purposes, and usually present with malaria infection upon their return to the district [3].

More than 10 years ago, Trenggalek district was a hyperendemic area with high transmission of malaria. There has been malaria outbreaks in 2006, 2007 and 2008 in three villages [4], but the malaria cases then decreased in 2012 to 2013, from 326 to 155 cases [5]. Recently, the malaria situation has changed significantly. Trenggalek district became a hypoendemic area with mainly imported cases brought by migrant workers from outside islands. This was demonstrated by the data in the third consecutive years in 2014, 2015 and 2016, when 77, 91, 52 malaria cases, respectively, were reported and all were imported cases. Until April 2017, there were 10 cases reported, all of which were imported cases [6]. The last suspected indigenous patient was found in 2012 with fever in Pandean Primary Health Centre (PHC) of Dongko, but was microscopically negative. Since then, malaria cases in the district were always imported cases [7] of migrant workers returning from outside islands. The present study aims to determine the existence of indigenous malaria cases and local transmission of malaria in the Trenggalek district.

The discovery of asymptomatic malaria carriers in former migrant workers of some villages of Pandean PHC was very surprising. Villagers looked healthy, but malaria parasites were found on their blood films. Asymptomatic malaria cases are not listed in the malaria data of health centre, as they did not feel sick and did not seek any medication [8]. Parasitaemia in asymptomatic malaria usually is very low [9] and can reach submicroscopic level that are unlikely to be detected by a well-trained microscopists [10, 11]. Submicroscopic malaria, therefore, potentially plays role as a silent reservoir [10], and can contribute to disease transmission [11], especially if suitable *Anopheles* mosquitoes exist in the environment.

## Methods

### Study site

The survey was done in a village of Salamwates, Dongko subdistrict, Trenggalek district, East Java Province. Trenggalek district is situated on the South-West of Surabaya, the capital city of East Java Province. This district is located at 111°24′–112°11′ east longitude and 7°63′–8°34′ south latitude, and bordered by Tulungagung district to the east, Indonesian Ocean to the south, Pacitan and Ponorogo districts to the west, Ponorogo and Tulungagung districts to the north. The district is a tropical region with dry and rainy seasons. The rainy season occurs in September–April, while the dry season in May–August. Average rainfall is 17.4 mm/year. The anomaly in climate causes a prolonged rainy season. Salamwates village belongs to field of activity of Pandean Primary Health Centre (PHC) and located on the highest plain in hilly area of Dongko subdistrict, which reaches 848 m above sea level with an average rainfall of 11 mm/year. The total population of Trenggalek district is 762,853 and the majority of them work as subsistence farmers. The village where the study was conducted includes an area of 12.63 km^2^ with total population of 7252 [12].

### Physical examination and blood sampling

The adult villagers aged from 18 years old were all invited to the house of the head of the village and were asked their consent for malaria blood screening. Upon written informed consent, they will be examined physically for any malaria symptoms. The data on age, sex, history of travel to malaria endemic areas, and history of malaria infection were also recorded. The travel history was needed to trace whether the infection was imported or local. Physical examination included measurement of body temperature and existing clinical symptoms. One mL of blood was drawn by venipuncture to prepare thin and thick blood films for parasite detection and identification, and dried blood spots on filter paper for polymerase chain reaction (PCR) amplification.

### Microscopic examination of blood films

Microscopical detection and identification of malaria parasites was carried out on Giemsa-stained thin and thick blood films. Detection was declared negative when as many as 100 fields with 100× objective magnification had been examined, but no parasites were found [13]. Examination was performed by two experienced field microscopists independently and re-read by third microscopist at the Laboratory of Parasitology, Faculty of Medicine, Universitas Airlangga.

### Parasite species identification from blood samples by PCR

Identification of parasite species was performed using a single-step PCR with slight modification. Briefly, DNA was isolated from blood spot using Qiagen DNA isolation kit (Qiagen, Tokyo-Japan) according to the manufacturer’s protocol. DNA was then stored at – 80 °C until used. Single step PCR was targetted against the 18S small subunit ribosomal RNA (ssu rRNA) gene of *Plasmodium vivax* and *Plasmodium falciparum.* Briefly, 5 µL of DNA template were mixed with PCR master mix (Biorad, Singapore) and the three primers in one tube. Primers and PCR condition were as described [14]. Single step PCR resulted in 266 bp of band that is specific for *P. vivax* and 346 bp specific for *P. falciparum.* Other species were not targetted in this research, since only those two species were always recorded for years in study area [7].

### Mosquito collection and species identification

Mosquito collection was done during dry season in June 2015. The method of collection was as described [15]. Adult mosquitoes were caught using human bait indoor. A volunteer villager stayed inside a double net-trap that consisted of an inner net of 1.2 × 1.2 × 2 m and outer net of 3.2 × 3.2 × 2 m. Mosquitoes which land on nets were caught using mouth-operated aspirator. In addition, human landing catch (HLC) was conducted hourly from 18.00 to 24.00 o’clock with 15 min rest, following previous experience where the vast majority of the *Anopheles* came to the house at this time. Captured mosquitoes were put into containers and labelled. Identification of mosquito species was carried out in the Department of Parasitology, Faculty of Medicine, Universitas Airlangga, using key identification [16, 17].

### Sporozoite detection in *Anopheles* mosquito

After the wings and legs were removed, DNA was isolated from the head and thoracal parts using Qiagen DNeasy isolation kit (Qiagen, Tokyo, Japan) following the manufacturer’s protocol. Primers used for PCR amplification was based on small subunit ribosomal RNA and PCR conditions used for parasite species identification from *Anopheles* mosquito were same as those used to identify the parasites from blood [16].

## Results

### Demographic characteristic of the subjects

Total number of subjects participated in this study were 64, consisted of 37 male and 27 female, and aged between 18 and 90 years old. The youngest participant was 18 years old. No subjects show clinical symptoms of malaria (Table 1). Other clinical symptoms were fever due to common cold, cough, back and joint pain, nausea and bloated stomach. Based on the history of malaria infection and travel to malaria endemic areas outside Java Island, the subjects were grouped into four groups; Group-1 consisted of 18 (28%) individuals who have previously been infected with malaria and have previously travelled to malaria endemic areas. Group-2 consisted of 2 (3.1%) persons who have previously been infected with malaria, but have never travelled to malaria endemic areas. Group-3 had 9 (14%) individual who have never been infected with malaria, but have previously travelled to malaria endemic areas. Group-4 consisted of 35 (54.7%) individuals who have never been infected with malaria and have not travelled to malaria endemic areas (Table 2).

**Table 1:** Characteristic of subject during blood sampling in Salamwates villages, Trenggalek district

	Number (%)	Total
Sex		
Male	37 (57.8)	64
Female	27 (42.19)	
Ages (years old)		
18–60	64 (100)	64
Clinical symptoms of malaria	None	
Other physical symptoms		
Fever	1 (1.56)	
Headache	32 (50.00)	
Nausea and bloated	14 (21.88)	
Tingling hands	2 (3.12)	
Stomagache, diarhoea	4 (6.25)	
Neck/nape pain	9 (14.06)	
Joint pain	8 (12.50)	
Back pain	1 (1.56)	
Itching	2 (3.12)	
Pale/anemic	12 (18.75)	
Splenomegaly	1 (1.56)

**Table 2:** History of malaria infection and traveling to endemic areas of the subjects in outside Java Island and microscopy examination

Group	History of malaria infection–traveling to endemic malaria areas	Total (%)	Species of *Plasmodium*	
			Number and species	Total (%)
1	Ever–ever	18 (28.1)	1 Pf, 2 mix	3 (4.69)
2	Ever–never	2 (3.1)	0	0 (0)
3	Never–ever	9 (14.1)	1 Pv	1 (1.57)
4	Never–never	35 (54.7)	2 Pf	2 (3.13)
	Total	64 (100)		6 (9.38)

Pf, *Plasmodium falciparum*; Pv, *P. vivax*; mix, Pf and Pv

### Microscopic detection of the blood films

Microscopic examination of 64 blood films showed that none of the subjects carried parasites. Analysis using PCR revealed 6 subjects (9.38%) were positive, 3 of which from the Group 1, where one subject was infected with *P. falciparum* and two subjects carried mixed infections of *P. falciparum* and *P. vivax.* Historically, all the three positive persons used to be infected with malaria and had travelled to malaria endemic areas in Kalimantan Island. One subject from the Group-3 was infected with *P. vivax*. The other two subjects infected with *P. falciparum*, were from Group-4 who had never previously been infected with malaria nor travelled to malaria-endemic areas (Table 2).

### *Anopheles* identification and sporozoite detection

**Fig. 1:**
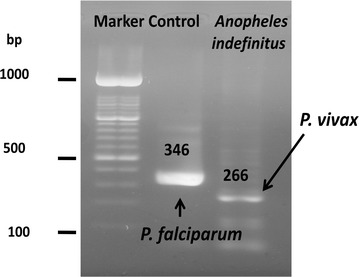
Species of *Plasmodium* from *An. indefinitus* was identified as *P. vivax* by single step PCR. The band of 266 bp was very clear compare with control of *P. falciparum* 3D7 strain from invitro culture (346 bp). Marker: 100 bp Invitrogen

Only one sample of *Anopheles* mosquito was caught and identified as *Anopheles indefinitus*. By PCR, it was found to carry parasite DNA of *P. vivax* (Fig. 1).

## Discussion

The Government of Indonesia in 2009 has set out a plan to eliminate malaria in the entire archipelago by 2030, with different timelines in each island according to malaria endemicity. The island of Java and Bali were set out to eliminate malaria in 2014 and are currently intensifying efforts to prevent re-introduction of malaria in districts that have been certified to be free of transmission. In such areas, active malaria surveillance with supervised radical cure for vivax cases, monitoring of migrant people and mapping of *Anopheles* vector were advised.

Trenggalek is the only district in East Java Province that has not been certified for malaria elimination due to the constantly reported malaria cases and, therefore, the study was conducted as a part of the efforts to assess the malaria transmission in the area. The district has reported no malaria cases in the PHC since 2014 except for the imported cases. This study also found no malaria cases in the microscopic screening of 64 subjects. However, further PCR analysis revealed a relatively high submicroscopic malaria cases (9.38%). Submicroscopic malaria cases have been reported in many studies previously [7, 24]. Based on the previous history of malaria infection and travel to malaria endemic areas, it is highly likely that all six cases, particularly with those with *P. falciparum,* were originated through indigenous transmission.

Submicroscopic and asymptomatic malaria have been reported, not only in high transmission regions in Ghana [18], Kenya [19], Thailand [20] and Bangladesh [21], but also in hypoendemic and low transmission of malaria, such as in Solomon Island [22] and Uganda [23]. Numerous submicroscopic infections reported, both in low [9, 22, 23] and high [24] endemic settings, in adults only [9, 24] or in all age groups [25], and involved *P. falciparum, P. vivax* [9, 25], *Plasmodium malariae*, *Plasmodium ovale* [26], and *Plasmodium knowlesi* [27]. The prevalence of submicroscopic malaria infection varied widely in each malaria region. Submicroscopic malaria is common in adults, in low endemic settings and in chronic infections [11]. The prevalence of submicroscopic malaria is > 5% in Ethiopia [26], and 72.9% in Solomon Islands [28], and may also occur in pregnancy [29]. Low-density of parasites in submicroscopic parasite carriage in hypoendemic areas can be a reservoir of infection and may contribute to sustaining transmission in areas with low or very low transmission intensity (under ~ 5%) [30], because they are gametocyte producers [9, 22, 29] and responsible of transmission.

In this study, the PCR-positive subjects were not treated as the diagnosis came late. Indeed, the untreated asymptomatic infection remains a major source of gametocytes for local mosquito vectors [31]. Management of submicroscopic malaria is currently still controversial as the treatment guideline still use laboratory confirmation either by microscopy or rapid diagnostic tests (RDT) as the point of care. Malaria infection with any density of parasite, asymptomatic or submicroscopic remains to be treated, because malaria infection have serious health and social consequences [32]. Drug treatment of asymptomatic and submicroscopic may be expected to increase the risk of clinical malaria upon reinfection [33], by interfering with immunity and trends to severe malaria [34]. In different studies, the duration of asymptomatic infection remained elusive [31]. The evidence from Colombia [35] and Peru [36] reported asymptomatic infection that cleared spontaneously within 14 and 7 days, respectively. In Africa, the duration of untreated submicroscopic infection in low endemic areas is even shorter [31].

The species of *An. indefinitus* was the only mosquito caught with the human bait collection method indoor and the single step PCR proved the parasite carriage of this species. This result confirmed that malaria transmission has occured locally, and suggests that *An. indefinitus* is a malaria vector in the study area. Specimen of *An. indefinitus* had also been caught in 2011, in the areas of Pandean PHC, Trenggalek district, where two mosquitoes of this species were found to rest on the wall inside a house. The research found that this species was dominantly zoophylic, since they were able to catch 94% of *An. indefinitus* with cow bait [37]. In district in West Java Province, a coastal area, *An. indefinitus* was caught both indoors and outdoors [38].

In this study, 2 of 6 submicroscopic malaria cases were found in individuals who had never previously been infected nor travelled to outside islands. Together with the analysis of the mosquito vector in the area, that confirms *An. indefinitus* as a vector of *P. vivax*, it was concluded that the submicroscopic malaria cases in Trenggalek district play a role in the local transmission of malaria. Apart from the findings presented, this study has several limitations, particularly the relatively small number of subjects who participated in this study, relative to the total population of the village, the difficulty to include children and the very limited number of *Anopheles* that could be collected.

In conclusion, malaria submicroscopic cases is relatively frequent in Trenggalek and this may be representative of many of the remaining malaria endemic foci in Java. Therefore, regular active malaria surveillance and supervised radical cure for vivax malaria cases, monitoring of the migrant workers, vector surveillance and vector-based interventions are mandatory to prevent reintroduction of malaria. Community participation either through village malaria workers to report visitors, or control of *Anopheles* breeding sites has also been demonstrated to contribute significantly to malaria elimination programmes.
